# Electronic Device Screen Time and Meibomian Gland Morphology in Children

**DOI:** 10.18502/jovr.v16i4.9741

**Published:** 2021-10-25

**Authors:** Özkan Kocamiş, Emine Temel, Nazife Aşikgarip, Kemal Örnek

**Affiliations:** ^1^Department of Opthalmology, Kırşehir Ahi Evran University School of Medicine, Kırşehir, Turkey

**Keywords:** Meibography, Meibomian Gland, Pediatric Age, SPEED Score

## Abstract

**Purpose:**

To investigate changes in meibomian gland morphology and impact of electronic device usage time on meibomian glands in pediatric age group.

**Methods:**

In this prospective study, 149 eyes of 149 children were enrolled. The participants also completed the Standard Patient Evaluation of Eye Dryness (SPEED) questionnaire and provided information regarding weekly hours spent in front of a digital screen. Meibography was performed in all subjects. Grading of images was evaluated using a previously validated 5-point meiboscale (0–4) for meibomian gland atrophy and a 3-point scale for meibomian gland tortuosity (0–2).

**Results:**

Of the 149 enrolled children, 83 (55.7%) were female and 66 (44.3%) male. The mean age was 13.0 ± 3.0 (range, 5–18) years. The mean loss of meibomian gland area was 20.80 ± 9.32%. The mean meiboscore was 1.20 ± 0.58 for gland atrophy and the mean tortuosity score was 0.99 ± 0.62. The mean screen time was 29.32 ± 16.18 hr/week. There was a weak and significantly positive correlation between loss of meibomian gland area and screen time (*r* = 0.210, *P* = 0.010). There was a weak and significantly positive correlation between meiboscore for gland atrophy and screen time (*r* = 0.188, *P* = 0.022). We found a weak but significantly positive correlation between meibomian gland tortuosity and screen time (*r* = 0.142, *P* = 0.033).

**Conclusion:**

Meibomian gland morphology may show changes in pediatric age group and excessive screen time may be a factor triggering these changes in gland morphology.

##  INTRODUCTION

Meibomian gland secretion plays a crucial role in maintaining ocular surface health. The lipid secretion of the meibomian glands is the main component of the outer layer of tear film and plays an important role in tear film stability.^[1]^ Meibomian gland dysfunction (MGD) is a chronic, diffuse abnormality of the meibomian glands, commonly characterized by duct obstruction and/or changes in the glandular secretion. Due to the inflammatory and obstructive nature of chronic MGD, meibomian gland atrophy may develop over time.^[2]^ Meibomian gland atrophy can be associated with a systemic disease or can be part of the normal aging process. It has been previously reported that the incidence of meibomian gland atrophy increases with age.^[1,3,4]^ However, little is known about the prevalence of meibomian gland atrophy in pediatric age group and there are few reports in the literature demonstrating alterations of meibomian glands in this population.^[5,6,7]^

The use of electronic devices (smartphones, tablets, and computers) has increased rapidly during the past decade. The long-term ocular effects of these devices are unknown. However, a range of short-term ocular surface discomfort as well as visual discomfort have been reported.^[8,9,10]^ Ocular surface discomfort includes dryness, stinging, burning, itchiness, and irritation of the eye. Visual discomfort includes blurred vision, difficulty refocusing when shifting focus from one distance to another, headache, and eye strain. Moon et al reported that smartphone use in children was strongly associated with pediatric dry eye disease.^[11]^

First described by Arita et al, noncontact infrared meibography is a relatively new technique used to obtain information about the morphologic characteristics of the meibomian glands.^[1,6,12]^ It is a noninvasive, rapid, and patient-friendly examination method that visualizes the silhouette of meibomian glands through infrared illumination of the everted eyelid from the conjunctival side.^[1,12]^

In this study, we aimed to investigate the meibomian gland morphology in pediatric age group and the effect of electronic device usage time on meibomian glands.

##  METHODS

In this prospective study, 149 eyes of 149 patients who presented to the University Hospital Outpatient Clinic for routine eye examination were included. Informed consent was obtained from parents of all participants in accordance with the Institutional Review Board Committee's approval and adherence to the declaration of Helsinki.

The participants under the age of 18 who could cooperate with image acquisition of meibomian glands were included. Exclusion criteria were history of dry eye disease, presence of MGD, presence of allergic conjunctivitis, contact lens use, past ocular surgery, ongoing medication use, and presence of any ocular or systemic disease.

Biomicroscopic slit-lamp examination was performed in all subjects. The participants also completed the Standard Patient Evaluation of Eye Dryness (SPEED) questionnaire and provided information regarding the number of hours per week spent in front of a digital screen.

The upper eyelid was everted and noncontact meibography was performed in all subjects using corneal topographic device (Sirius, CSO, Florence, Italy) with Phoenix-Meibography Imaging software module. Only right eyes were included in the study. The morphological characteristics of the meibography images were evaluated by blinded physicians.

**Figure 1:**
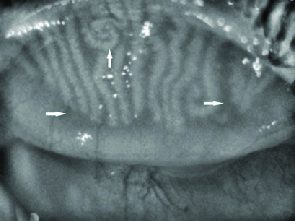
Horizontal arrows show atrophic areas and vertical arrow shows increasing tortuosity.

Previously validated 5-point meiboscale (0–4) for meibomian gland atrophy was used to grade the images.^[13]^ Meiboscale is as follows: grade 0: normal meibomian glands, grade 1: ≤ 25% gland atrophy, grade 2: 26–50% gland atrophy, grade 3: 51–75% gland atrophy, and grade 4: > 75% gland atrophy [Figure 1]. Grading of meibomian gland tortuosity was also performed using a 3-point scale (0–2) with tortuosity defined as a > 45-degree angle of the meibomian gland; grade 0: no distortion, grade 1: 1–4 glands distorted, and grade 2: ≥ 5 glands distorted [Figure 1].^[14]^

**Figure 2:**
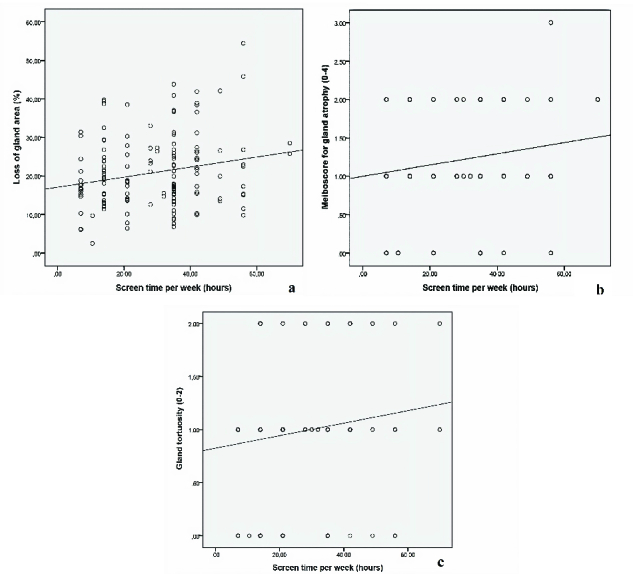
(a) Correlation graph of loss of meibomian gland area and screen time. (b) Correlation graph of meiboscale for gland atrophy and screen time. (c) Correlation graph of meibomian gland tortuosity and screen time.

The first noninvasive tear film break-up time (NIF-BUT) and average noninvasive tear film break-up time (NIBUT) were also evaluated by the software for each case.

All statistical analyses were performed using IBM SPSS 15.0 (SPSS Inc., Chicago, IL, USA). Testing for normal distribution was performed by Kolmogorov–Smirnov test. The mean and standard deviation values were provided for numerical variables with a normal distribution. For numerical variables with normal distribution, the difference between the two groups was determined by *t*-test in independent groups. The Pearson's correlation coefficient was used to measure the strength of a linear association between two variables, where the value *r* = 1 means a perfect positive correlation, and the value *r* = –1 means a perfect negative correlation. Statistical significance was defined at a level of 5% (*P* < 0.05).

##  RESULTS

Of the 149 children included in the study, 83 (55.7%) were female and 66 (44.3%) male. The mean age was 13.0 ± 3.0 (range, 5–18) years. The mean loss of meibomian gland area was 20.80 ± 9.32%. The mean meiboscale was 1.20 ± 0.58 for gland atrophy and the mean tortuosity score was 0.99 ± 0.62. One-hundred thirty-seven (91.4%) participants had evidence of meibomian gland atrophy (meiboscale ≥ 1) and 120 (80.5%) had evidence of meibomian gland tortuosity (tortuosity score ≥ 1). Demographics and clinical characteristics of the participants are summarized in Table 1.

**Table 1:** Demographics and clinical characteristics for all participants


**Sex ** ***n*** ** (%) **	
Female	83 (55.7)
Male	66 (44.3)
**Age (yr) **	
Mean ± SD	13.0 ± 3.0
(range)	(5–18)
**Meiboscore for gland atrophy ** ***n*** ** (%) **	
Grade 0	12 (8.05)
Grade 1	95 (63.7)
Grade 2	41 (27.5)
Grade 3	1 (0.67)
Grade 4	–
**Tortuosity score ** ***n*** ** (%) **	
Grade 0	29 (19.5)
Grade 1	92 (61.7)
Grade 2	28 (18.8)
SD, standard deviation; yr, year	

**Table 2:** Gender distribution of study parameters


**Parameter**	**Females**	**Males**	***P*** **-value**
Screen time (hr) per week (Mean ± SD)	30.03 ± 15.74	26.68 ± 13.95	0.177
Loss of gland area (%) (Mean ± SD)	21.22 ± 9.15%	20.26 ± 9.57%	0.538
Meiboscore for gland atrophy (Mean ± SD)	1.25 ± 0.53	1.15 ± 0.63	0.294
Tortuosity score (Mean ± SD)	1.07 ± 0.57	0.89 ± 0.65	0.081
NIF-BUT (s) (Mean ± SD)	11.94 ± 5.42	10.91 ± 5.41	0.247
Average NIBUT (s) (Mean ± SD)	12.5 ± 4.81	11.2 ± 5.25	0.112
SD, standard deviation; NIF-BUT, noninvasive first tear film break-up time; NIBUT, noninvasive tear film break-up time *Statistically significant *p*-value			

### SPEED Score

The average SPEED score was 0.93 ± 3.55 with a range from 0 to 20. No significant association was found between the SPEED score and loss of meibomian gland area (*P* = 0.236), meiboscale for gland atrophy (*P* = 0.218), neither for gland tortuosity (*P* = 0.596).

### Noninvasive Tears Film Break-up Time

The mean NIF-BUT was 11.49 ± 5.42 s ranging from 2 to 17 s. The average NIBUT was 11.94 ± 5.04 s with a range from 2 to 17 s.

### Screen Time

The mean screen time was 29.32 ± 16.18 hr/week and ranged from 7 to 70 hr. There was a weak and significantly positive correlation between the loss of meibomian gland area and screen time (*r* = 0.210, *P* = 0.010). There was a weak and significantly positive correlation between meiboscale for gland atrophy and screen time (*r* = 0.188, *P* = 0.022). We found a weak and significantly positive correlation between meibomian gland tortuosity and screen time (*r* = 0.142, *P* = 0.033). Correlation graphs of meibomian gland parameters and screen time are shown in Figure 2.

### Subgroup Analysis By Gender

The mean screen time per week was 30.03 ± 15.74 hr in females and 26.68 ± 13.95 hr in males (*P* = 0.177). The mean loss of meibomian gland area was 21.22 ± 9.15% in females and 20.26 ± 9.57% in males. There was no statistically significant difference between genders in terms of loss of meibomian gland area (*P* = 0.538). The mean meiboscale for gland atrophy was 1.15 ± 0.63 in males and 1.25 ± 0.53 in females. The mean tortuosity score was 0.89 ± 0.65 in males and 1.07 ± 0.57 in females. No statistically significant difference was found between genders for atrophy and tortuosity (*P* = 0.294 and *P* = 0.081, respectively).

The average NIBUT was 12.5 ± 4.81 s in females and 11.2 ± 5.25 s in males (*P* = 0.112). The mean NIF-BUT was 11.94 ± 5.42 s in females and 10.91 ± 5.41 s in males (*P* = 0.247). The distribution of parameters in each gender is presented in Table 2.

##  DISCUSSION

In the current study, we assessed alterations of meibomian gland morphology in pediatric population. We also investigated the correlation between electronic device usage time with the parameters measured.

Alterations in meibomian gland morphology are considered to be related to glandular function disorders. Recent studies have shown that gland dropout occurs as an age-related atrophic process.^[1,2]^ The prevalence of MGD is known to be significantly increased in older age groups. In this study, although the enrolled pediatric population had no history of dry eye disease or MGD, approximately 91.4% showed some evidence of meibomian gland atrophy. This is a higher rate compared to previous studies. Gupta et al evaluated the prevalence of meibomian gland atrophy and gland tortuosity in a pediatric population.^[13]^ They showed that 42% of the subjects had evidence of meibomian gland atrophy.^[15]^ This observation suggests that meibomian gland atrophy is a common condition and can be found in asymptomatic pediatric population.

The question at this point is unveiling the causative factors in meibomian gland atrophy in the pediatric population. Increasing numbers of dry eye disease and MGD in the general population suggest that environmental factors may trigger this condition in patients with subclinical MGD. Considering that pediatric cases are less symptomatic than adults, long-term outcomes can be improved with regular follow-ups in pediatric patients at risk.

In a study by Gupta et al, it was shown that 42% of the subjects had evidence of meibomian gland atrophy and 37% had evidence of meibomian gland tortuosity.^[15]^ They did not find an increase in tortuosity in 63% of the cases. In our study, 137 subjects (91.4%) had some degree of meibomian gland atrophy and 120 (80.5%) had some degree of meibomian gland tortuosity. We did not find an increase in tortuosity in 19.5% of the cases. Gupta et al also reported that the mean meiboscale was 0.58 ± 60.80 for gland atrophy and 0.45 ± 0.64 for tortuosity.^[14]^ According to our results, the mean meiboscale was 1.20 ± 0.58 for gland atrophy and the mean tortuosity score was 0.99 ± 0.62. Wu et al divided their participants into two groups: child (age range, 3 to 11 years) and adolescent (age range, 12 to 18 years) groups.^[1]^ They reported that meibomian gland loss was observed in both groups, but the meiboscale was not significantly different between the two groups.^[1]^

In addition, we found that changes in meibomian gland morphology were not significantly different between males and females. Contrary to our findings, Mizoguchi et al evaluated the morphology and function of meibomian glands in junior high school students at 15 years of age and reported that changes to the meibum quality were significantly greater in males than in females.^[14]^ According to the authors, the differences may be due to sex hormones like androgens and estrogens. Both androgens and estrogens were previously shown to affect meibomian gland function.^[16,17,18,19,20,21,22]^

In this study, children with a previous diagnosis of DED were excluded and the SPEED questionnaire was used to screen for symptoms suggestive of MGD and dry eye. Based on the criteria reported by Asiedu et al,^[22]^ the majority of our study population fell into the asymptomatic group. Therefore, the results of our study support that there may be atrophy in the meibomian glands in pediatric population even in asymptomatic subjects. Han et al have shown that children with objective clinical findings of dry eye disease, similar to those seen in adult patients, tend to be less symptomatic compared to adults.^[23]^ This demonstrates the importance of follow-up visits for MGD in children who are at risk of developing ocular surface disease.

Today, the population is increasingly more dependent on their digital devices like computers, tablets, and smartphones. This also affects the pediatric population as they spend excessive time on laptops and tablet devices at school and at home. Long-term visual display terminal work, especially for more than 4 hr daily, was associated with a high incidence of dry eye disease.^[25]^ Fenga et al reported that the development of ocular complaints in visual display terminal workers was correlated with MGD.^[25]^ MGD has been generally considered the main reason for evaporative dry eye. The use of electronic devices that make people keep their eyes widely open has been recognized to decrease the frequency of blinking and accelerate tear evaporation. Moon et al evaluated the risk factors of dry eye disease in school children associated with video display terminal use.^[26]^ Their results showed that smartphone use is an important dry eye disease risk factor in children. Moon et al and Gupta et al did not find a significant correlation between the amount of time spent in front of a visual display device and meibomian gland atrophy.^[13,26]^ According to our findings, the risk of developing atrophy and tortuosity in meibomian glands is higher in children when screen time is increased.

Our study has some limitations. The first is the inability to completely observe the meibomian glands in the pediatric age group due to poor compliance in children. Everting the upper eyelid made the examination process very difficult. Second, the studied population was from a single tertiary care center, which might have resulted in selection bias (all patients were living in a city with a population of 100,000).

In summary, there was a significant, albeit weak, relationship between the time spent in front of screens and the meibomian gland changes in pediatric age group. In today's digital information age, it is recommended for children to be followed and monitored for meibomian gland changes.

##  Financial Support and Sponsorship

Nil.

##  Conflicts of Interest

There are no conflicts of interest.
